# Association between serum lactate dehydrogenase and frailty among individuals with metabolic syndrome

**DOI:** 10.1371/journal.pone.0256315

**Published:** 2021-09-03

**Authors:** Li-Hsiang Chen, Li-Wei Wu

**Affiliations:** 1 Division of Family Medicine, Department of Family and Community Medicine, Kaohsiung Armed Forces General Hospital, Kaohsiung, Taiwan, Republic of China; 2 Division of Family Medicine, Department of Family and Community Medicine, Tri-Service General Hospital; and School of Medicine, National Defense Medical Center, Taipei, Taiwan, Republic of China; 3 Division of Geriatric Medicine, Department of Family and Community Medicine, Tri-Service General Hospital, Taipei, Taiwan, Republic of China; 4 Health Management Center, Department of Family and Community Medicine, Tri-Service General Hospital, National Defense Medical Center, Taipei, Taiwan, Republic of China; 5 Graduate Institute of Medical Sciences, National Defense Medical Center, Taipei, Taiwan, Republic of China; University of California Irvine, UNITED STATES

## Abstract

While metabolic syndrome (MetS) is associated with frailty, the correlation of serum lactate dehydrogenase (sLDH) and frailty with MetS remain uncertain. To investigate the relationship between sLDH and frail components in the US with MetS. A total of 4,066 participants aged 40–90 years were assessed from the database of the third National Health and Nutrition Examination Survey, 1988–1994. The participants were classified into MetS and non-MetS groups. Multivariate logistic regression analysis with four models were performed to assess the odds ratio (OR) of the divided tertiles of sLDH levels with frailty, and frail components including slow walking (SW), weakness, exhaustion, low physical activity (LPA), and low body weight (LBW). Higher sLDH levels were positively associated with frailty in the MetS group (*p* = 0.024) but not in non-MetS group (*p* = 0.102). After covariate adjustments, the OR of frailty in the upper two tertiles compared to the lowest tertile and revealed statistical significance (*p* < 0.05). Frail components of SW, weakness, exhaustion, and LPA were associated with higher sLDH (*p* < 0.05) except for LBW in MetS and non-MetS groups. The results demonstrated the strong association of higher sLDH levels and frailty among US individuals with MetS.

## Introduction

LDH is an indispensable enzyme for glycolysis in cytoplasmic anaerobic metabolism, which catalyzes the interconversion of the reduced form of nicotinamide adenine dinucleotide (NADH) to the oxidized form of NAD+, and pyruvate to lactate, producing the adenosine triphosphate (ATP) as the energy for human [1–4]. The five LDH isoforms consist of homomeric and dimeric tetramers of LDHA (= LDH-M, M) and LDHB (= LDH-H, H) subunits (i.e. LDH1 = 4H, LDH2 = 3H1M, LDH3 = 2H2M, LDH4 = 1H3M, LDH5 = 4M). In skeletal muscle LDH5 and in the heart LDH1 is predominant [1, 2, 5]. LDH exists in various tissues such as the brain, skeletal muscle, heart, lung, liver, pancreas, red blood cells, and kidney [5]. The enhanced sLDH signifies the pathological conditions of acute tissue or cellular damage [1, 5]. Previous studies demonstrated that elevated sLDH were associated with multiple health problems, including osteoporosis, congestive heart failure (CHF), chronic pulmonary obstructive disease (COPD), hypoxia, cirrhosis of the liver, inflammatory disease, human immunodeficiency disease, and malignancy [3, 4, 6–8]. Moreover, higher sLDH enhanced the cardiovascular mortality of individuals with chronic arsenic exposure in Taiwan and increased the risk of all-cause mortality in US populations with MetS [2, 3, 9].

According to the criteria of the Third Report of National Cholesterol Education Program Adult Treatment Panel III (NCEP-ATP III), MetS is a constellation of various metabolic disorders, including obesity, dyslipidemia, hyperglycemia, and hypertension [10]. The clusters of metabolic abnormalities substantially increase the risk of insulin resistance (IR), type 2 diabetes mellitus (DM), CVD, inflammation, and oxidative stress (OS), which accelerates the aging process [10–12]. Individuals aged more than 40 years increase the risk of incident MetS in the NHANES cohort [11, 13, 14]. The average rate of MetS calculated from approximately 24.3% to 39.1% globally [11, 13, 14]. The Longitudinal Aging Study Amsterdam with 1,247 participants reported MetS was associated with a 19-year all-cause mortality [15].

Frailty is a geriatric syndrome of decreased physical function. On the basis of the investigation with the Cardiovascular Health Study (CHS) by Fried et al., the original concepts of frailty included three or more of five phenotypes: shrinking (unintentional weight loss), weakness (declined grip strength), exhaustion, slowness, and low activity [16]. According to the theories of cycle of frailty, the mutual exacerbation of negative energy balance, sarcopenia, nutritional deficiency, inflammation, and neuroendocrine dysregulation increase vulnerability to environmental stressors in frail subjects; these issues in turn, lead to a risk of impaired physiological homeostasis, disability, falls, cognitive disorders, chronic diseases, hospitalization, and mortality [16–19]. Older age, malnutrition, smoking, alcohol consumption, CVD, DM, and multiple comorbidities are all more prevalent in frail people [16, 17, 20, 21]. Aging, lack of physical activity (PA), chronic diseases, adverse lifestyle, and nutrient imbalance make a close link between frailty and MetS in adults [11, 19, 22–25]. The prevalence of frail individuals 60 years or older ranged from 2% to 34%, compared to various population-based studies [16, 18, 22, 26, 27]. Kane *et al*. speculated that frailty in older adults with MetS had been estimated to be about 45.5% by the NHANES database in 2003~2006 [28]. Frailty and MetS correlate with comorbidities and adverse health outcomes for the elderly, whereas few literatures have discussed frailty and MetS in middle-aged populations. One research enrolled 10,020 participants aged 20 years and older addressed the association between frailty, MetS and mortality [28]. A cohort study of 493,737 participants aged 37–73 years exhibited the relationship between frailty, multimorbidity, specific long-term conditions, and mortality [29]. sLDH may act as a predictive biomarker reflecting the impacts of hemostatic dysregulation. Yuichi Nakazato *et al*. pointed out that frail patients who underwent maintenance hemodialysis significantly correlated with elevated sLDH [30]. However, the concepts of correlation between LDH levels, frailty, and MetS have not been elucidated. Therefore, we aim to explore the association between sLDH and frailty among middle-aged and older US populations with MetS by NHANES III datasets.

## Materials and methods

### Ethics statement

The execution of the NHANES III was approved in accordance with the protocol of the Institutional Review Board by National Center for Health Statistics of the Centers for Disease Control and Prevention (CDC). All eligible participants were carefully informed of the benefits and risks, and commenced the survey with informed consents. All procedures performed in this study were in accordance with the ethical standards of the institutional research committee and with the 1964 Helsinki declaration and its later amendments.

### Study designs and subjects

The enrolled data was obtained from the NHANES III between 1988 and 1994, which constituted complete laboratory samples, physical examinations, questionnaires for multistaged and stratified investigations of the US population by home examinations, household interviews and mobile examination centers (MECs) [31]. The study was composed of 4,066 US participants from 40 to 90 years, classified in two groups of 2,268 individuals with MetS and 1,798 individuals with non-MetS. The demographic characteristics, laboratory tests, physical function, and mental conditions were analyzed thoroughly, and complied with the NHANES III protocol. Data with unavailable relevance for sLDH, frailty, and MetS were excluded from our study.

### Definition of MetS

Participants diagnosed with MetS met at least three of the five identification markers, based on criteria of NCEP-ATP III [10]: (1) abdominal obesity with waist circumference > 102 cm in men and > 88 cm in women; (2) triglyceride (TG) ≥150 mg/dL; (3) high-density lipoprotein cholesterol (HDL-C) < 40 mg/dL in men and < 50 mg/dL in women; (4) impaired plasma fasting glucose ≥ 110 mg/dL (6.1 mmol/L) or current DM; (5) elevated systolic blood pressure (SBP) ≥ 130 mmHg or DBP ≥ 85 mmHg.

### Construction of frailty

Frailty as syndrome has been widely clarified in a variety of theories, with clinical phenotypes by Fried or the FI by Rockwood *et al*. [16, 17]. In this article, frailty was defined as three or more of the following components based on modifications of phenotypic criteria constructed by Wilhelm-Leen *et al*. [27] in NHANES III cohorts: (1) SW, defined as the slowest quintile using the 8-foot gait speed test adjusted for gender; (2) Weakness, defined as the answer of “some difficulty,” “much difficulty,” or “unable to do” from participants with the question of how much difficulty they had while “lifting or carrying something as heavy as 10 pounds (like a sack of potatoes or rice).”; (3) Exhaustion, defined as the answer of “some difficulty,” “much difficulty,” or “unable to do” from participants with the question of how much difficulty while “walking from one room to the other on the same level.”; (4) LPA, defined as the answer of “less active” from participants with the issue being “compared with most (men/women) your age, would you say that you are more active, less active, or about the same?”; (5) LBW was, defined as BMI ≤ 18.5 *kg*/*m2*.

### Definition of the LDH tertiles

Serum LDH levels were defined as reference values of 0–210 U/L and abnormalities > 210 U/L according to the laboratory tests results reporting criteria from NHANES datasets [31]. The evaluation of sLDH levels (65–668 U/L) in our study included normal and abnormal values. To verify the correlation between LDH and frailty with MetS, the examinations of LDH were plotted into three tertiles based on the research by Wu *et al*. using the LDH tertiles to explore the significant association between sLDH and all-cause mortality with MetS [3]. The reference group was regarded as the lowest tertile for all participants. The cut-off points for serum LDH tertiles were T1 (65–149), T2 (149–176) and T3 (176–668) U/L.

### Covariates measurements

The demographic information was obtained from participants in NHANES III, including age, gender, race/ethnicity, smoking status, medical history (congestive heart failure (CHF), stroke, asthma, malignancy, and type 2 DM), and PA. BMI was measured by dividing a person’s weight in kilograms with the square of height in meters (*kg*/*m2*). The average values of SBP and DBP were calculated after three or four times records with a mercury sphygmomanometer in the MEC and home survey. Hypertension was defined by the average BP ≥ 140/90 mmHg of a self-reported physician’s diagnosis. DM was defined by either the examinations of serum fasting glucose level ≥ 126 mg/dl, random glucose level ≥ 200 mg/dl, current diabetic medication use, or a self-report of a medical diagnosis. The biochemical values used various analyzers and instrumentation at the Lipoprotein Analytical Laboratory of Johns Hopkins University, Baltimore, Maryland from 1988 to 1994. All calculated metrics were performed with standardized methods and were compatible with the accurate reference protocol from the CDC. The participants were asked about the frequency of PA, which they did in their leisure time for the most recent month, which included swimming, dancing, jogging, walking, running, riding a bike, callisthenic exercise, resistance training, gardening, or other sports; however, the duration of PA in the US population was not evaluated in NHANES III (1988–1994). On the basis of intensity thresholds of metabolic equivalent tasks (METs), accessing the metabolic ratio of energy expense during activity to resting status, people were defined by three types, which differed with the engaged levels of PA weekly: (1) Ideal type (physically active), defined as an individual’s PA reaching the METs between 3 and 6, performing 5 or more times, or the METs more than 6 with performing 3 or more times, or moderate intensity for more than 150 minutes, or vigorous intensity for more than 75 minutes, or moderate and vigorous intensity for more than 150 minutes; (2) Intermediate type, defined as an individual’s PA, was between active and none, or moderate intensity was between 1 and 149 minutes, or vigorous intensity was between 1 and 74 minutes, or moderate and vigorous intensity was between 1 and 149 minutes; (3) None type (physically inactive), was defined as an individual with poor PA.

### Mortality and follow-up data

According to a probabilistic match between NHANES III participants older than 17 years and National Death Index (NDI) death certificate records, the mortality status, and follow-up data were obtained from the publicly available NHANES III Linked Mortality File by National Center for Health Statistics. Person-months of follow-up data on mortality of the NHANES III survey participants recorded from 1988 to December 31, 2006 [32].

### Statistical analysis

All statistical parameters were analyzed by the Statistical Package for the Social Sciences (SPSS, Inc., Version 18 for Windows, Chicago, IL, USA). The mean and standard deviation (SD) were measued for quantitative analysis, whereas numbers and percentages were measured for qualitative analysis. The tests for continous and discrete variables included the Wilcoxon Rank sum test, the independent t-test, and the Chi-square test, respectively. Statistical significance was asserted as two-sided *p*-values of less than 0.05. The association between sLDH and frail components in MetS populations was performed by multivariate logistic regression. An extensive-model design was applied for covariate adjustments for potential confounding factors as follows: model 1 = unadjusted; model 2 = adjusted by model 1 + age, sex, race, BMI; model 3 = adjusted by model 2 + SBP, serum TGs, CRP, total bilirubin, and glucose levels; model 4 = adjusted by model 3 + PA, smoking, CHF, stroke, asthma, and malignancy.

## Results

### Preliminary study population characteristics

Among the 4,066 participants from 40 to 90 years in the present study, 2,268 participants had MetS, while 1,798 participants did not have MetS. The analytical parameters and demographic characteristics in the NHANES III datasets were available for this research. The mean age of MetS groups was 71.93 ± 7.99 years old, and 44.5% were male. The mean age of non-MetS groups were 71.00 ± 7.94 years old, and 56.5% were male. The comparisons of participants in MetS and non-MetS groups with frailty and without frailty are summarized in Table 1. Participants with MetS in the frailty group were prone to higher sLDH levels, older age, higher body mass index (BMI), lower diastolic pressure (DBP), higher serum TGs, lower serum total bilirubin, higher serum uric acid, higher serum glucose, and higher serum total protein (all, *p* <0.05). However, there were no significant correlations between higher sLDH levels and frailty in the non-MetS group (*p* = 0.102). In both MetS and non-MetS groups, those with frailty were inclined to CHF, stroke, type 2 DM, lack of PA, whereas malignancy displayed nonsignificant findings in MetS (*p* = 0.861) and non-MetS groups (*p* = 0.617).

**Table 1:** Characteristics of the study participants with MetS and non-MetS with and without frailty.

Variables	Metabolic syndrome		Total *N* = 2268	*P-*value	Non-metabolic syndrome		Total *N* = 1798	*P-*value
	Non-frailty group N = 2119	Frailty group *N* = 149			Non-frailty group *N* = 1714	Frailty group *N* = 84		
**Continuous Variables** ^a^								
Serum LDH (U/L), mean (SD)	173.28 (43.82)	181.67 (42.76)	173.84 (43.79)	0.024	167.28 (39.23)	174.51 (45.94)	167.62 (39.58)	0.102
Age (years), mean	71.73 (7.95)	74.64 (8.09)	71.93 (7.99)	<0.001	70.88 (7.88)	73.45 (8.81)	71.00 (7.94)	0.004
(SD) BMI(kg/m2), mean (SD)	28.54 (4.91)	30.99 (6.75)	28.70 (5.08)	<0.001	25.03 (4.05)	25.58 (6.55)	25.06 (4.20)	0.247
Systolic BP (mmHg), mean (SD)	147.81 (21.75)	146.21 (22.04)	147.70 (21.77)	0.392	133.68 (23.43)	135.73 (25.85)	133.78 (23.54)	0.446
Diastolic BP (mmHg), mean (SD)	74.09 (13.94)	71.20 (15.69)	73.90 (14.08)	0.017	70.42 (13.81)	68.72 (13.81)	70.34 (13.81)	0.278
Serum triglycerides (mg/dL)	164.67 (112.49)	207.30 (166.38)	167.47 (117.22)	<0.001	157.32 (100.04)	143.57 (96.68)	156.67 (99.90)	0.218
Serum cholesterol (mg/dL), mean (SD)	221.69 (45.07)	224.44 (47.89)	221.87 (45.25)	0.473	224.41 (43.49)	220.00 (47.95)	224.20 (43.70)	0.367
Serum LDL-cholesterol (mg/dL), mean (SD)	141.04 (38.34)	136.94 (33.41)	140.83 (38.10)	0.446	140.84 (41.18)	132.29 (48.93)	140.50 (41.50)	0.285
Serum HDL-cholesterol (mg/dL), mean (SD)	48.48 (16.16)	51.18 (17.84)	48.65 (16.28)	0.051	54.40 (16.13)	60.57 (18.78)	54.69 (16.31)	0.001
Serum CRP (mg/dL), mean (SD)	0.61 (0.97)	0.74 (0.82)	0.62 (0.96)	0.111	0.49 (1.01)	0.59 (0.72)	0.49 (0.99)	0.374
Serum total bilirubin (umol/L), mean (SD)	0.59 (0.30)	0.53 (0.29)	0.58 (0.30)	0.034	0.60 (0.29)	0.53 (0.33)	0.60 (0.29)	0.021
Serum uric acid(mg/dL)	5.81 (1.53)	6.13 (1.76)	5.83 (1.54)	0.015	5.55 (1.52)	5.38 (1.56)	5.54 (1.52)	0.338
Serum glucose (mg/dL), mean (SD)	119.16 (48.64)	140.25 (70.94)	120.54 (50.66)	<0.001	96.96 (28.93)	97.40 (29.85)	96.98 (28.97)	0.890
Serum total protein (g/dL), mean (SD)	7.35 (0.48)	7.46 (0.53)	7.35 (0.49)	0.008	7.28 (0.48)	7.38 (0.58)	7.28 (0.48)	0.067
Serum AST(U/L), mean (SD)	21.73 (12.79)	21.26 (9.60)	21.70 (12.60)	0.662	21.81 (8.99)	21.00 (8.42)	21.77 (8.96)	0.421
Serum ALT (U/L), mean (SD)	15.00 (11.08)	13.96 (8.68)	14.93 (10.94)	0.262	13.83 (8.12)	12.90 (7.95)	13.78 (8.12)	0.310
**Categorical Variables** ^b^								
Male, N(%)	970 (45.8)	39 (26.2)	1009 (44.5)	<0.001	981(57.2)	35 (41.7)	1016 (56.5)	0.005
Non-Hispanic white, N(%)	1228 (58.0)	67 (45.0)	1295 (57.1)	0.020	1074 (62.7)	35 (41.7)	1109 (61.7)	0.001
Congestive heart failure, *N* (%)	176 (8.3)	27 (18.1)	203 (9.0)	0.001	122 (7.1)	16 (19.0)	138 (7.7)	<0.001
Stroke, *N* (%)	139 (6.6)	28 (18.8)	167 (7.4)	<0.001	79 (4.6)	17 (20.2)	96 (5.3)	<0.001
Asthma, *N* (%)	134 (6.3)	12 (8.1)	146 (6.4)	0.684	104 (6.1)	10 (11.9)	114 (6.3)	0.032
Malignancy, *N* (%)	152 (7.2)	12 (8.1)	164 (7.2)	0.861	156 (9.1)	9 (10.7)	165 (9.2)	0.617
Type 2 diabetes mellitus, *N* (%)	428 (20.2)	58 (38.9)	486 (21.4)	<0.001	115 (6.7)	14 (16.7)	129 (7.2)	0.003
Smoker, *N* (%)	357 (16.8)	15 (10.1)	372 (16.4)	0.031	316 (18.4)	10 (11.9)	326 (18.1)	0.129
Physical activity, *N* (%)				<0.001				<0.001
Ideal	674 (31.8)	29 (19.5)	703 (31.0)		650 (37.9)	12 (14.3)	662 (36.8)	
Intermediate	913 (43.1)	22 (14.8)	935 (41.2)		719 (41.9)	14 (16.7)	733 (40.8)	
None	532 (25.1)	98 (65.8)	630 (27.8)		345 (20.1)	58 (69.0)	403 (22.4)	

Abbreviation: N: number; SD: standard deviation; LDH: lactate dehydrogenase; BMI: body mass index; SBP: systolic blood pressure; DBP: diastolic blood pressure; LDL: low-density lipoprotein; HDL: high-density lipoprotein; AST: aspartateaminotransferase; ALT: alanine aminotransferase; CRP: C-reactive protein.

Values a interpreted as mean (standard deviation).

Values b interpreted as number (%).

### Association between Frailty and LDH

The association between frailty and tertiles of sLDH levels in adults with and without MetS are shown in Table 2. The utilization of multivariate logistic regression to approach the comparisons in four models demonstrated that participants with frailty had significantly higher sLDH levels in the MetS group (all, *p* < 0.05) after we adjusted for potential confounding variables in model 2, model 3, and model 4. The OR of the highest tertile in the adjusted models 2, 3 and 4 compared to the lowest tertile in the unadjusted model 1 were 2.08 (95% CI 1.12 to 3.84; *p* = 0.02), 2.21 (95% CI 1.19 to 4.11; *p* = 0.012), 2.45 (95% CI 1.32 to 4.56; *p* = 0.005), respectively. There was no significant association between increased LDH tertiles and frailty in the non-MetS group. Table 3 exhibited the relationship between frail components and LDH tertiles in four models of the MetS and non-MetS groups. Among the five components of frailty, SW, weakness, exhaustion, and LPA, we found positive associations with MetS in the highest LDH tertiles after adjustments for all covariates in all models (*p* <0.05). Moreover, weakness, exhaustion, and LPA showed statistical significance in the non-MetS group (*p* < 0.05) and in the MetS group. The OR of the comparison between the highest and lowest tertile of LBW revealed statistical significance only in model 1, at 2.70 (95% CI 1.17 to 6.25; *p* = 0.020), in the MetS group and 1.82 (95% CI 1.29 to 2.57; *p* = 0.001), and in the non-MetS group, respectively. After adjusting for all covariates, there was no statistical significance in models 2, 3, and 4 in both MetS and non-MetS subjects.

**Table 2:** Association between frailty and serum LDH level in study participants with MetS and non-MetS.

Models^a^ Tertiles		OR (95% CI)	*P*Value	OR (95% CI)	*P* Value
		Metabolic syndrome group		Non-metabolic syndrome group	
**Model 1**	T2 v.s. T1	2.09 (1.12, 3.90)	0.021	1.22 (0.60, 2.48)	0.577
	T3 v.s. T1	2.65 (1.44, 4.87)	0.002	1.57 (0.80, 3.08)	0.187
**Model 2**	T2 v.s. T1	2.13 (1.14, 3.99)	0.018	1.00 (0.49, 2.06)	0.998
	T3 v.s. T1	2.08 (1.12, 3.84)	0.020	1.11 (0.56, 2.20)	0.768
**Model 3**	T2 v.s. T1	2.13 (1.14, 4.00)	0.018	1.05 (0.50, 2.18)	0.898
	T3 v.s. T1	2.21 (1.19, 4.11)	0.012	1.06 (0.52, 2.14)	0.880
**Model 4**	T2 v.s. T1	2.33 (1.23, 4.39)	0.009	0.86 (0.41, 1.82)	0.693
	T3 v.s. T1	2.45 (1.32, 4.56)	0.005	1.02 (0.52, 2.18)	0.872

Tertiles of serum lactic dehydrogenase level: T1 (65–149 U/L), T2 (149–176 U/L) and T3 (176–668 U/L).

^a^Adjusted covariates.

Model 1 = Unadjusted.

Model 2 = adjusted by Model 1 + (age, sex, race, BMI).

Model 3 = adjusted by Model 2 + (systolic blood pressure, serum triglycerides, serum C-reactive protein, serum total bilirubin and glucose).

Model 4 = adjusted by Model 3 + (physical activity, smoking, congestive heart failure, stroke, asthma and malignancy).

Abbreviation: OR: odds ratio; CI: confidence interval.

**Table 3:** Association between frail components and serum LDH level in study participants with MetS and non-MetS.

Models ^a^ Tertiles		OR (95% CI)	*P* Value	OR (95% CI)	*P* Value
		Metabolic syndrome		Non-metabolic syndrome	
**Slow walking**					
**Model 1**	T2 v.s. T1	1.42 (1.01, 2.01)	0.046	1.30 (0.85, 1.99)	0.220
	T3 v.s. T1	2.10 (1.52, 2.92)	0.000	1.56 (1.04, 2.35)	0.033
**Model 2**	T2 v.s. T1	1.49 (1.05, 2.10)	0.025	1.14 (0.74, 1.75)	0.548
	T3 v.s. T1	1.80 (1.29, 2.51)	0.000	1.22 (0.81, 1.84)	0.350
**Model 3**	T2 v.s. T1	1.52 (1.07, 2.15)	0.020	1.12 (0.73, 1.72)	0.619
	T3 v.s. T1	1.84 (1.31, 2.57)	0.000	1.10 (0.72, 1.68)	0.665
**Model 4**	T2 v.s. T1	1.53 (1.08, 2.18)	0.018	1.08 (0.70, 1.66)	0.745
	T3 v.s. T1	1.95 (1.39, 2.73)	0.000	1.10 (0.72, 1.68)	0.654
**Weakness**					
**Model 1**	T2 v.s. T1	1.30 (1.02, 1.65)	0.037	1.52 (1.16, 1.99)	0.002
	T3 v.s. T1	1.81 (1.43, 2.28)	0.000	2.08 (1.61, 2.71)	0.000
**Model 2**	T2 v.s. T1	1.27 (0.99, 1.62)	0.056	1.49 (1.14, 1.95)	0.004
	T3 v.s. T1	1.58 (1.25, 2.01)	0.000	1.74 (1.34, 2.27)	0.000
**Model 3**	T2 v.s. T1	1.28 (1.00, 1.63)	0.050	1.48 (1.13, 1.94)	0.004
	T3 v.s. T1	1.56 (1.23, 1.98)	0.000	1.73 (1.32, 2.25)	0.000
**Model 4**	T2 v.s. T1	1.33 (1.04, 1.70)	0.023	1.35 (1.02, 1.77)	0.033
	T3 v.s. T1	1.70 (1.34, 2.16)	0.000	1.63 (1.25, 2.12)	0.000
**Exhaustion**					
**Model 1**	T2 v.s. T1	1.62 (0.98, 2.68)	0.061	1.55 (0.91, 2.64)	0.106
	T3 v.s. T1	2.15 (1.33, 3.48)	0.002	2.32 (1.40, 3.84)	0.001
**Model 2**	T2 v.s. T1	1.64 (0.99, 2.72)	0.055	1.49 (0.87, 2.54)	0.143
	T3 v.s. T1	1.86 (1.14, 3.03)	0.013	1.91 (1.15, 3.18)	0.013
**Model 3**	T2 v.s. T1	1.64 (0.99, 2.72)	0.056	1.46 (0.86, 2.50)	0.164
	T3 v.s. T1	1.80 (1.10, 2.94)	0.020	1.86 (1.12, 3.11)	0.018
**Model 4**	T2 v.s. T1	1.69 (1.02, 2.81)	0.044	1.28 (0.75, 2.20)	0.367
	T3 v.s. T1	1.98 (1.21, 3.24)	0.007	1.73 (1.03, 2.90)	0.038
**Low physical activity**					
**Model 1**	T2 v.s. T1	1.12 (0.91, 1.39)	0.297	1.25 (1.01, 1.54)	0.038
	T3 v.s. T1	1.54 (1.25, 1.89)	0.000	1.83 (1.48, 2.26)	0.000
**Model 2**	T2 v.s. T1	1.07 (0.86, 1.32)	0.565	1.21 (0.98, 1.49)	0.081
	T3 v.s. T1	1.36 (1.10, 1.67)	0.005	1.65 (1.33, 2.06)	0.000
**Model 3**	T2 v.s. T1	1.08 (0.87, 1.33)	0.507	1.22 (0.99, 1.50)	0.069
	T3 v.s. T1	1.34 (1.08, 1.66)	0.007	1.65 (1.32, 2.05)	0.000
**Model 4**	T2 v.s. T1	1.09 (0.88, 1.35)	0.418	1.09 (0.88, 1.35)	0.448
	T3 v.s. T1	1.39 (1.13, 1.73)	0.002	1.51 (1.21, 1.88)	0.000
**Low body weight**					
**Model 1**	T2 v.s. T1	1.55 (0.63, 3.82)	0.336	1.57 (1.14, 2.17)	0.006
	T3 v.s. T1	2.70 (1.17, 6.25)	0.020	1.82 (1.29, 2.57)	0.001
**Model 2**	T2 v.s. T1	0.40 (0.14, 1.16)	0.093	0.98 (0.70, 1.38)	0.911
	T3 v.s. T1	1.15 (0.45, 2.96)	0.771	1.01 (0.69, 1.48)	0.943
**Model 3**	T2 v.s. T1	0.35 (0.12, 1.01)	0.052	0.98 (0.70, 1.39)	0.920
	T3 v.s. T1	0.88 (0.32, 2.43)	0.800	1.00 (0.67, 1.47)	0.981
**Model 4**	T2 v.s. T1	0.40 (0.14, 1.19)	0.101	0.95 (0.67, 1.35)	0.781
	T3 v.s. T1	1.16 (0.39, 3.48)	0.791	1.04 (0.71, 1.54)	0.833

Tertiles of serum lactic dehydrogenase level: T1 (65–149 U/L), T2 (149–176 U/L) and T3 (176–668 U/L).

aAdjusted covariates.

Model 1 = Unadjusted.

Model 2 = adjusted by Model 1 + (age, sex, race, BMI).

Model 3 = adjusted by Model 2 + (systolic blood pressure, serum triglycerides, serum C-reactive protein, serum total bilirubin and glucose).

Model 4 = adjusted by Model 3 + (physical activity, smoking, congestive heart failure, stroke, asthma and malignancy).

Abbreviation: SW, slow walking speed; LPA, low physical activity; LBW, low body weight; OR, odds ratio; CI, confidence interval.

## Discussion

The remarkable findings of the present study indicate a significant association between higher sLDH levels and frailty in MetS. Moreover, sLDH was positively associated with frail components: SW, weakness, exhaustion, and LPA with MetS. The characteristics of frail individuals were more likely middle-age and the elderly, the female gender, obese, smokers, and those with a medical history of CHF, stroke, and type 2 DM. To the best of our knowledge, this is the first study to demonstrate a positive association between sLDH and frail components in US populations with MetS.

Prior articles have shown that most people with MetS and frailty were older, had multiple comorbidities, and LPA [15, 28, 33]. Aging plays a crucial role in enhancing the risk of frailty, MetS, and the clear disadvantages of health problems. The predisposing factors of aging include obesity, OS, IR, inflammation, and malnutrition [11, 12, 21]. Many detrimental effects of aging lead to mitochondrial dysfunction, malnutrition, sedentary behavior (SB), muscle weakness, diminished bone mass, metabolic abnormalities, and inflammation [12, 24, 33–36]. Multifactorial circumstances might interconnect aging, MetS, and frailty. As such, the OS caused by reactive oxygen species (ROS) influenced human physiological function and pathophysiology. The production of free radicals from aging, mitochondrial dysfunction, and MetS increase OS, which in turn accelerates the aging process and overwhelms the cellular homeostasis, leading to mitochondrial DNA mutation, tissue injury, inflammation, CVD, MetS, and neurodegenerative disorders [12, 37, 38]. Aging, OS, mitochondrial dysregulation, and MetS might inflict elevated sLDH and impaired ATP production for an individual’s energy, leading to a decline of muscular strength and mass, physical inactivity, generalized weakness, SW, and gradual development of frailty [12, 35, 37–39]. Under the conditions of age-linked MetS and OS, sLDH might have a close relationship with frailty. Our study elucidated increased OR of frailty as being associated with higher sLDH in MetS individuals after various covariate adjustments.

The measurements of sLDH have been clinically utilized as indicators of tissue damage, muscle fatigue, inflammatory disease, and multiple organ disease worldwide. The chronic inflammatory process may be a crucial factor for chronic disease and cancer. The common etiologies of chronic inflammation and disease due to adverse lifestyles are compromised by smoking, alcohol abuse, unhealthy diet (red meat, high fat, glucose, and calories), nutrition deficiencies, stress, and LPA [36, 40, 41]. MetS is considered a chronic inflammatory state [9, 11]. Emerging evidence has demonstrated that obesity is associated with IR, type 2 DM, and chronic inflammation, especially in skeletal muscle inflammation [11, 42, 43]. Previous researches have exhibited that miscellaneous connections between obesity-derived IR and skeletal muscle enhanced the inflammatory process and impairment of myocytes and adipocytes [9, 11, 39, 42]. The dysregulation of adipose tissue, owing to IR and chronic inflammation, results in decreased glucose uptake, endothelial dysfunction, and metabolic disorder, thus facilitating the reduction of age-associated skeletal muscle mass (MM) [9, 11, 39, 42]. The loss of skeletal MM and declining MS increase the risks of frailty and mortality in the elderly [33, 35]. A cross-sectional study of 512 subjects aged 60 years or older using datasets of the NAMGARAM-2 cohort by Yoo *et al*. [43] showed female population over 65 years old with low handgrip strength showed a higher prevalence of osteoporosis and lower gait speed than normal-hand grip strength. Among frail components, the most common presentation of this symptom initially was weakness and could become a predictor for the onset of frailty [18]. Moreover, MetS was also an independent variable with weakness and viewed as a predictor of changes in frailty status of declining-hand grip strength and gait speed with longitudinal follow-up [21].

Inflammation plays a primary factor for frailty. Elevated plasma inflammatory markers inclusive of IL-6, white blood cells (WBC), TNF-α, CRP, and fibrinogen have been noted in the relationship with frailty [44, 45]. LDH was regarded as a biomarker for inflammation [4, 46], and consequences of MetS-related inflammatory processes cause a number of biological disorders, contributing to the elevated sLDH and increased risk of frailty. Inflammation could be an additional explanation for the association of higher sLDH with frailty in people with MetS.

The state of exhaustion is related to the interaction of physical and mental fatigue. Frail individuals are inclined to have depressive symptoms versus those without frailty [26, 44]. The presence of exhaustion, accompanied with MetS-related IR, may cause the presence of SB and LPA in middle-aged or older frail populations. Prior studies have shown that a sedentary lifestyle with LPA increased the risk of chronic disease, MetS, mortality from cardiometabolic, and all-cause diseases [25, 34, 47, 48]. Meanwhile, there were higher serum hydroxyproline, LDH, creatine kinase, troponin I, lower calcium, and 25(OH)D levels among the elderly with LPA of SB, which accelerated deconditioning, muscle fatigability, declined MM, MS, and antioxidant capacity [49]. This supported the result of our study regarding the relationship between higher sLDH and frailty with MetS.

Unhealthy dietary lifestyle and malnutrition in older adults increase the risks for a cluster of adverse health problems such as inflammation, declining antioxidant function, sarcopenia, obesity, or lower BMI, MetS with IR, cancer, mortality, and frailty [20, 21]. Frail subjects with MetS are prone to have nutritional deficiency, and disturb homeostasis and physical function. Therefore, malnutrition is potentially positively associated with elevated sLDH level among frail populations with MetS. Despite LBW showing a nonsignificant correlation with elevated sLDH in all adjusted models, the higher sLDH showed increasing trends to the other four frail components, thus fulfilling the criteria of frailty and could address the relationships. People with underweight or obesity significantly increased the risk of frailty than those with normal weight [22, 26]. Among frail individuals with MetS, higher fat mass reduced the metabolic rate and physical performance, resulting in weight gain; as such, the obvious decrease in weight might not occur rapidly but turned into LBW until assessing long-term follow-up. We concluded that higher sLDH levels can be regarded as independent predictors of frailty in those with clinical MetS.

There were several limitations in this article. First, the secondary analysis of the NHANES III versus persistent long-term follow-up restricted the causal inference. Second, the bias of results might have been contributed from the measurement of sLDH only once, without repeatedly collected data during periods of follow-up. The correlation of serum LDH levels collected only at a single point is a drawback for the study. Third, phenotypes of frailty relied on self-report and individual examinations. Inappropriate manifestations and reported bias might occur if participants with acute illness at that time. The direct assessments of the handgrip test, sit and walk, and other validated tests did not include in the article. Fourth, the comparisons between LDH and frail components were assessed by frail phenotypes, which limited the outcomes in consideration of other factors among frailty index (FI) [17] or various measurements, as well as the different identification of participants with MetS by the criteria of NCEP-ATP III or International Diabetes Federation and cut-off points of LDH levels that might cause unpredictable findings. Fifth, the trend of LBW among frail patients with MetS (under the influence of LDH) needs more evidence to demonstrate results. Sixth, the under measurement of residual confounders should be assessed, as they might have potential effects on the association between LDH and frailty, despite adjusting for confounding factors. Activity of specific types of the LDH isoform, linked with frailty in those with MetS, needs rigorous investigation and research. Finally, the sLDH level impacting frailty only in people with MetS but not in the non-MetS group requires substantial evidence to prove the argument.

In conclusion, the present study highlighted higher serum LDH level had a significant association with frailty in middle-aged and older US populations with MetS. LDH was positively associated with frail phenotypes particularly in slow walking, weakness, exhaustion, and low physical activity. LDH might act as a predictive biomarker for frail people with MetS, whereas the correlation between elevated LDH and LBW or other causes of frailty beyond our discussion in patients with MetS remained controversial. The issues focused on the precise biological mechanisms underlying the molecular pathway and the correlation of higher serum LDH and frailty; we look forward to assessing this in further studies.

## Abbreviations

MetS: metabolic syndrome
sLDH: serum lactate dehydrogenase
US: United States
NHANES: National Health and Nutrition Examination Survey
SW: slow walking
LPA: low physical activity
LBW: low body weight
BMI: body mass index
SBP: systolic blood pressure
DBP: diastolic blood pressure TGs: triglycerides
CRP: C-reactive protein
NADH: nicotinamide adenine dinucleotide
ATP: adenosine triphosphate
CHF: congestive heart failure
COPD: chronic pulmonary obstructive disease
NCEP-ATP: National Cholesterol Education Program Adult Treatment Panel
IR: insulin resistance
DM: diabetes mellitus
CVD: cardiovascular disease
OS: oxidative stress
CHS: Cardiovascular Health Study
MEC: mobile examination center
CDC: Centers for Disease Control and Prevention
HDL-C: high-density lipoprotein cholesterol
LDL: low-density lipoprotein
AST: aspartateaminotransferase
ALT: alanine aminotransferase
kg/m2: weight in kilogram with the square of height in meter
METs: metabolic equivalent tasks
SD: standard deviation
OR: odds ratio
CI: confidence interval
MM: muscle mass
MS: muscle strength
BMD: bone mineral density
25(OH)D: 25-hydroxy vitamin D
ROS: reactive oxygen species
IL-6: interleukin 6
TNF-α: tumor necrosis factor alpha
VEGF: vascular endothelial growth factor
